# Tissue-type plasminogen activator suppresses activated stellate cells through low-density lipoprotein receptor-related protein 1

**DOI:** 10.1038/labinvest.2015.94

**Published:** 2015-08-03

**Authors:** Liang-I Kang, Kumiko Isse, Kelly Koral, William C Bowen, Selen Muratoglu, Dudley K Strickland, George K Michalopoulos, Wendy M Mars

**Affiliations:** 1 Department of Pathology, University of Pittsburgh School of Medicine, Pittsburgh, PA, USA; 2 Center for Vascular and Inflammatory Diseases, University of Maryland School of Medicine, Baltimore, MD, USA; 3 Department of Physiology, University of Maryland School of Medicine, Baltimore, MD, USA; 4 Department of Surgery, University of Maryland School of Medicine, Baltimore, MD, USA

## Abstract

Hepatic stellate cell (HSC) activation and trans-differentiation into myofibroblast (MFB)-like cells is key for fibrogenesis after liver injury and a potential therapeutic target. Recent studies demonstrated that low-density lipoprotein receptor-related protein 1 (LRP1)-dependent signaling by tissue-type plasminogen activator (t-PA) is a pro-fibrotic regulator of the MFB phenotype in kidney. This study investigated whether LRP1 signaling by t-PA is also relevant to HSC activation following injury. Primary and immortalized rat HSCs were treated with t-PA and assayed by western blot, MTT, and TUNEL. *In vitro* results were then verified using an *in vivo*, acute carbon tetrachloride (CCl_4_) injury model that examined the phenotype and recovery kinetics of MFBs from wild-type animals *vs* mice with a global (t-PA) or HSC-targeted (LRP1) deletion. *In vitro*, in contrast to kidney MFBs, exogenous, proteolytically inactive t-PA suppressed, rather than induced, activation markers in HSCs following phosphorylation of LRP1. This process was mediated by LRP1 as inhibition of t-PA binding to LRP1 blocked the effects of t-PA. *In vivo*, following acute injury, phosphorylation of LRP1 on activated HSCs occurred immediately prior to their disappearance. Mice lacking t-PA or LRP1 retained higher densities of activated HSCs for a longer time period compared with control mice after injury cessation. Hence, t-PA, an FDA-approved drug, contributes to the suppression of activated HSCs following injury repair via signaling through LRP1. This renders t-PA a potential target for exploitation in treating patients with fibrosis.

Liver cirrhosis is a growing global health concern due to an increased prevalence of chronic liver diseases, including viral hepatitis and non-alcoholic fatty-liver disease. Regardless of etiology, chronic parenchymal injury leads to accumulation of scar tissue and organ dysfunction through a highly orchestrated process centered on the emergence of a *de novo* population of myofibroblast (MFB)-like cells. Hepatic stellate cells (HSCs) are considered one of the major sources of matrix-depositing MFB-like cells in chronic liver injury. In response to inflammatory cytokines and growth factors, HSCs become activated and exhibit increased proliferative, migratory, contractile, and fibrogenic phenotypes.^1^ Understanding the factors that regulate HSC activation and trans-differentiation is the key to designing effective therapies.

The plasminogen activators (PAs) are multi-functional serine proteases involved in fibrinolysis, cellular migration,^2^ growth factor activation,^3^ and hepatic repair.^4, 5^ Loss of PAs delays liver regeneration after acute injury; this has been largely attributed to sustained fibrin deposition and loss of growth factor and matrix metalloproteinase activation.^4^ However, the roles of PA in chronic liver injury have been ambiguous,^6, 7^ possibly due to their pleiotropic functions on multiple cell types.

Emerging literature indicates that the biological effects of PAs in select systems are not limited to their proteolytic function. In fact, signaling functions may be equally, if not more, important to clearly understand their biological roles. In particular, the tissue-type PA (t-PA) is a key endogenous signaling molecule in injury and disease. One prominent t-PA signaling receptor is LRP1 (low-density lipoprotein receptor-related protein 1). LRP1 is a multi-ligand receptor often associated with protease–inhibitor complex and growth factor clearance; however, following ligand binding it can signal to promote cellular migration, differentiation, and changes in viability or proliferation.^8^

Recent studies involving kidney fibroblasts have defined a role for LRP1 in modulating tissue fibrosis and the MFB phenotype.^9^ As HSCs are now known to express LRP1,^10, 11^ and little is known about the role of LRP1 in HSCs during liver injury and regeneration, this study sought to establish whether t-PA affects fibrosis through LRP1 in HSCs. We anticipated a pro-fibrotic response, similar to kidney; however, here, we instead present data supporting an anti-fibrotic role for t-PA in liver, identifying it as a potential therapeutic for treating fibrosis.

## MATERIALS AND METHODS

### Reagents and Antibodies

Recombinant single-chain (American Diagnostica, Stanford, CT, USA; Molecular Innovations, Novi, MI, USA) or an inhibitor-treated, non-proteolytic form of human t-PA (FPRCK-TPA, Molecular Innovations) was used for cell-culture experiments. FPRCK-TPA is tested to have zero enzymatic activity by functional assay (personal communication with the company). Antibodies used for immunohistolabeling and Western blotting include: *α*-SMA (Clone 1A4; Dako, Carpinteria, CA, USA; Sigma-Aldrich, St Louis, MO, USA), p-LRP1 (Santa Cruz Biotechology, Santa Cruz, CA, USA), LRP1 (Clone 11H4; ATCC, Manassas, VA, USA; #3501, American Diagnostica), Collagen I (Abcam, Cambridge, MA), fibrinogen (Molecular Innovations), p-Akt (Thr308) and total Akt (both Cell Signaling Technology, Danvers, MA, USA), p-ERK1/2 and total ERK1/2 (Cell Signaling Technology). The PI3K pathway inhibitor LY294002 was from Cell Signaling Technology. Human RAP was prepared as previously described.^12^

### Rat HSC Isolation and Cell Culture

Male Fischer 344 rats (Charles River, Wilmington, MA, USA) ≥200 g in weight were used to isolate primary HSCs via an adaptation from Riccalton-Banks, *et al.*^13^ Briefly, liver cells were dissociated using an *in situ* two-step collagenase-perfusion method.^14, 15^ After low-gravity centrifugation of total liver cell suspension to pellet hepatocytes, non-parenchymal cells (NPC) were isolated from supernatants. The NPC fraction was washed once in complete medium (Dulbecco's Modified Eagle Medium+10% fetal bovine serum+0.1% gentamicin solution) and plated on uncoated six-well tissue-culture plates. Cells were maintained in a humidified incubator at 37 °C with 5% CO_2_. Complete medium was replaced 24 h post-plating. At 48 h post-plating, cells were serum-starved for 24 h before treatments. HSC cultures were minimally 80–85% pure. Rat HSC-T6 and human LX-2 HSC cell lines (Dr Scott Friedman, Mt. Sinai School of Medicine, New York, NY, USA) were maintained in the same medium as primary HSCs. For experiments, HSC-T6 cells were seeded at 3 × 10^5^ cells/ml, incubated overnight and then serum-starved for 24 h prior to treatment. LX-2 cells were seeded at 1 × 10^5^ cells/ml and grown to ~60% confluence prior to serum starvation and subsequent treatment. NRK-49F cells (ATCC) were cultured in Dulbecco's modified Eagle's medium:Ham's F12 (1:1) supplemented with 5% FBS until ~70% confluent, then serum-starved 24 h prior to treatments as indicated. All experiments were performed in serum-free conditions at 37 °C in a humidified incubator. All experiments were performed in replicate (two or more times), using pooled samples to obtain sufficient protein or RNA. Actual *n* for each replicate experiment performed is indicated in the legend.

### MTT Assay

HSC-T6 cells were seeded at 3 × 10^5^ cells/ml and incubated overnight in complete medium. After washing with serum-free medium, cells were treated with vehicle control or t-PA (10 nM) for 48 h serum-free. The MTT-based *in vitro* toxicology assay kit (Sigma-Aldrich) was used according to manufacturer instructions.

### TUNEL Assay

HSC-T6 cells were seeded and treated as described for the MTT assay. After 48 h, cells were fixed in 1% paraformaldehyde and labeled as indicated using the ApopTag Peroxidiase *In Situ* Apoptosis Detection Kit (Millipore, Temecula, CA, USA). A blinded team member assessed staining. Minimally, 10 low-powered images (× 100 magnification) were quantified for each condition.

### SDS-PAGE and Western Blot

Cell cultures were collected in lysis buffer (10 mM Tris buffer with 1% sodium dodecyl sulfate and protease/phosphatase inhibitor cocktail, defined as follows: Sigma-Aldrich catalog numbers: P8340, P2714, P2850, and P5726 (at recommended dilutions), AEBSF (50 *μ*g/ml), and sodium amiloride (1 mmol/l) and analyzed by western blotting as previously described.^16^ Briefly, equal amounts of protein (~30 *μ*g) from each sample was mixed with loading buffer with or without 100 mmol/l dithiothreitol, heated to 65 °C for 15 min, resolved by electrophoresis on 8 or 10% SDS-polyacrylamide gels, and transferred to polyvinylidene difluoride membranes for western blot analyses. Membranes were blocked in 5% milk or fish gelatin in Tris-buffered saline+Tween, followed by incubation with primary antibody in 5% blocking buffer. Horseradish peroxidase-conjugated secondary antibodies (Jackson ImmunoResearch Laboratories, West Grove, PA, USA) were used at a concentration of 1:50 000. Blots were developed using enhanced chemiluminescence substrate (Pierce, Rockford, IL, USA) and visualized on X-ray film. Loading equivalence was assessed from densitometry of scanned images of Ponceau S (0.2% solution) staining performed immediately after transfer of protein onto the polyvinylidene difluoride membranes (see Statistical Analysis section for more detail). Protein concentration of samples was assessed by the bicinchoninic acid assay (Thermo Fisher Scientific, Rockford, IL, USA). Primary antibody concentrations used were as follows: *α*-SMA (1:500–1:2000), p-LRP1 (1:500), LRP1 (1:1000–1:2000), PDGFR*β* (1:500), p-Akt (1:1000) and total Akt (1:1000), p-ERK1/2 (1:1000), and total ERK1/2 (1:1000).

### Immunoprecipitation

HSC-T6 cells were treated with vehicle control or t-PA for 1 min and lysed in ice-cold CHAPS buffer (10 mM CHAPS, 20 mM HEPES (pH 7.4), 150 mM NaCl, 2 mM CaCl_2_) supplemented with a phosphatase and protease inhibitor cocktail, as described above (Sigma-Aldrich). To obtain sufficient protein, multiple wells were pooled for these experiments.

One hundred micrograms of lysate from each treatment were incubated with the monoclonal antibody against LRP1 (11H4) or IgG control at 4 °C overnight with rotation. Protein A/G PLUS agarose beads (Santa Cruz Biotechnology) were added to the lysates and incubated at 4 °C for 3 h. The beads were pelleted, washed with CHAPS buffer, and the bound proteins were extracted from the beads in reducing sample buffer. Proteins were resolved on 10% SDS-polyacrylamide gel, and analyzed by western blotting as described above, using anti-phospho-tyrosine (1:250; BD Biosciences, San Jose, CA, USA) and anti–LRP1 (1:1000; 11H4) antibodies.

### Reverse Transcription and Polymerase Chain Reaction

The primer sequences used (as previously described by Li *et al.*^17^ and Vogel *et al.*^18^) were as follows: Collagen I Forward Primer 5′-AAC GGC AAG GTG TTG TGC CAT G-3′, Collagen I Reverse Primer 5′-AGC TGG GGA GCA AAG TTT CCT C-3′, *β*-actin Forward Primer 5′-GAG CTA TGA GCT GCC TGA CG-3′, *β*-actin Reverse Primer 5′-GTG CTA GGA GCC AGG GCA GTA A-3′. Total RNA was isolated from treated cells using RNABee (Amsbio, Lake Forest, CA, USA)-chloroform extraction and precipitated with isopropanol. Isolated total RNA was DNase I-treated using Turbo DNase kit (Life Technologies, Grand Island, NY, USA) according to the manufacturer's instructions. First-strand cDNA was synthesized from DNAse I-treated RNA using SuperScript III reverse transcriptase (Life Technologies) with random hexamers. One hundred nanograms of cDNA from each sample were used for polymerase chain reaction to amplify type I *α*1-collagen gene (*Col1a1*) *or β*-actin gene (*Actb*). Negative control reactions were run for each primer set in which reverse transcriptase or cDNA template were not added to ensure for specificity of reactions and lack of contamination, respectively (data not shown). PCR consisted of 30 cycles at 94 °C for 1 min, 59 °C for 1 min, and 72 °C for 1 min, followed by a final extension step at 72 °C for 7 min. PCR products were run on a 2% agarose gel containing ethidium bromide and visualized under UV light for photography.

### Carbon Tetrachloride (CCl_4_) Acute Injury and Resolution Experiments

Male C57Bl/6 and t-PA^−/−^ animals (Jackson Laboratories, Bar Harbor, ME, USA), or *LRP*^*flox/flox*^*;SM22-cre*^+/−^ mice (Strickland laboratory, University of Maryland, Baltimore, MD, USA), aged 10–12 weeks, were given 0.05% phenobarbital (PB) water *ad libitum* for 1 week prior to corn oil (control; data not shown) or CCl_4_ intraperitoneal injections (1 *μ*l/g body weight, diluted 1:4 with corn oil for injection; Sigma-Aldrich). Two acute doses of CCl_4_ were given 3 days apart and mice were collected from 1–14 days after injection (*n*=3–5 for days 1–7; *n*=2 for day 14). Others have used these conditions, with PB specifically utilized as a means of equalizing CCl_4_ bioactivation.^19^ During the course of the CCl_4_ experiments, animals were housed in filter-top cages with unlimited water and standard chow placed in outside-vented chemical hoods to limit CCl_4_ exposure up to day 5 after CCl_4_ injury. These conditions were in accordance to the recommendations made by the Department of Environmental Health and Safety at the University of Pittsburgh. Total and liver body weights were recorded at the time of killing. Serum and liver tissues were collected for biochemical and histological analyses, with tissues fixed in formalin for paraffin embedding, frozen in OCT for immunofluorescence, and flash frozen in liquid nitrogen for protein and RNA extractions. The University of Pittsburgh Medical Center-Presbyterian Hospital, Department of Pathology Lab Support Services quantified serum alanine transaminase (ALT) levels.

### Immunohistochemical Staining and Fluorescent Labeling

Paraffin-embedded livers (*n*=3–5 per time point) were sectioned into 4-*μ*m thick slices. Standard immunohistochemistry was performed using anti-*α*-SMA (1:50), anti-Collagen I (1:100), and anti-fibrinogen (1:50) primary antibodies with an avidin–biotin complex-horseradish peroxidase developer. Double staining on sections used mouse anti-*α*-SMA (1:50) and rabbit anti-p-LRP1 (1:200) primary antibodies and Qdot-conjugated Q705 and Q605 secondary antibodies, respectively (Life Technologies). Triple staining was accomplished by adding goat anti-t-PA (1:150) labeled with biotinylated secondary and Streptavidin-Q655. Specificity of the *α*-SMA was confirmed using a mouse-on-mouse blocking kit (Vector Laboratories, Burlingame, CA, USA). Sequential double labeling of 4-*μ*m thick OCT sections from *LRP*^*flox/flox*^; SM22-cre ^+/− or −/−^, and t-PA^+/+ or −/−^ mice (1:1 acetone:MeOH fix) used anti-LRP1 (1:50, American Diagnostica) with a Cy5-conjugated secondary antibody, followed by Cy3-conjugated anti-*α*-SMA (1:1000, Sigma-Aldrich).

### Image Capture and Analysis

The Center for Biologic Imaging at the University of Pittsburgh provided Provis microscopes for brightfield (Olympus, Center Valley, PA, USA), Olympus Fluoview 1000 upright confocal microscopy for fluorescence, and Metamorph software (Sunnyvale, CA, USA) for quantification of positive-staining area and co-localization analyses. For immunohistochemical quantification, low-powered images (× 100 or × 200 total magnification) were taken at regular intervals to cover the majority of the tissue. Edges, large vessel lumens, and artifacts were avoided. Only slides that were stained and imaged simultaneously were compared. Images were thresholded for positive staining and batch analyzed for area of staining using the same threshold, expressed as a percentage of the total visual field analyzed. Qdot-labeled tissues were scanned with a Zeiss AxioVision MIRAX MIDI scanner (Carl-Zeiss, Jena, Germany) and single frames were captured with the Panoramic Viewer from 3DHISTECH (Budapest, Hungary).

### Statistical Analysis

Densitometry from scanned western blot and PCR gel images was analyzed using NIH ImageJ 1.42q (National Institutes of Health, USA). For western blots, protein loading and transfer efficiency were normalized using scanned lane images from Ponceau S-stained membranes, prior to immunoblotting,^20^ after verifying this is also a valid control in our hands (Supplementary Figure S1). Statistical analyses were performed using Prism (GraphPad, La Jolla, CA, USA). Numeric values are expressed as the ratio of the treated target/loading control divided by the untreated sample/loading control. Student's *t*-test and paired Student's *t*-test were used to compare two groups and two groups with normalized ratios, respectively. For more than two groups, one-way analysis of variance test was performed with Tukey's *post hoc* test analyses. Statistical significance was set at *P*<0.05, with variability presented as±s.e.m. Statistics were only performed when replicate experiments were run on the same gels due to potential artifacts from differences in exposure times.

### Animal Use Approval

Studies were approved by the University of Pittsburgh Institutional Animal Care and Use Committee in accordance to the ‘Guide for the Care and Use of Laboratory Animals' published by the National Institutes of Health. The University of Pittsburgh is an accredited institution by the Association for Assessment and Accreditation of Laboratory Animal Care.

## RESULTS

### Exogenous t-PA Decreases Markers of HSC Activation in Primary and Transformed Cells

**Figure 1:**
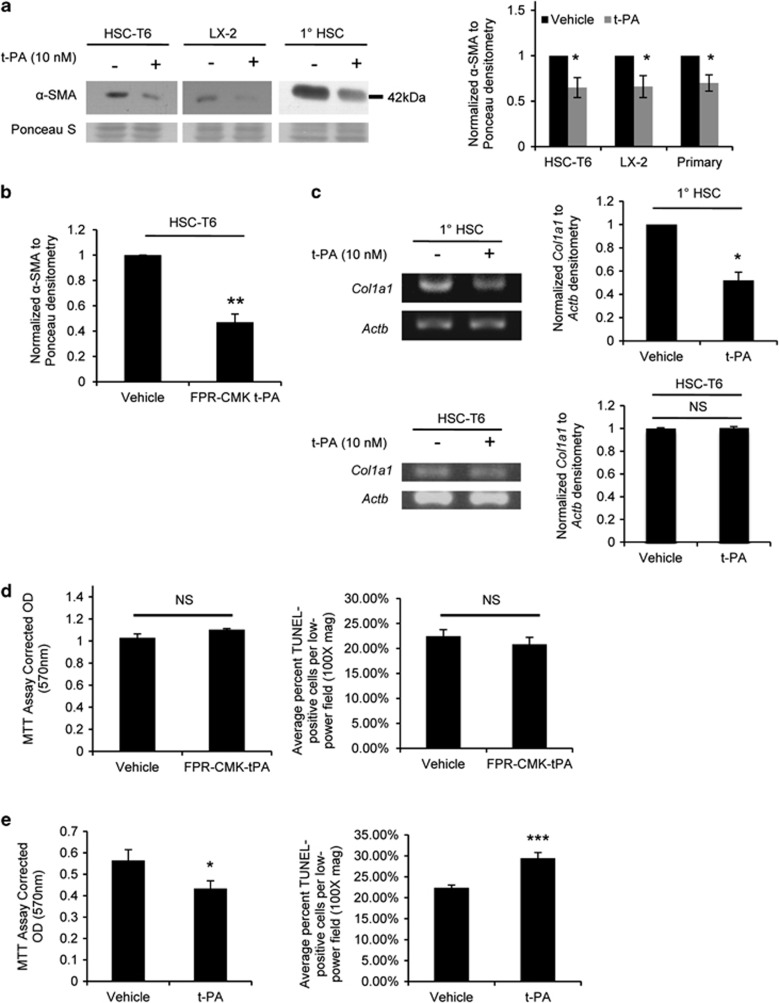
t-PA inhibits HSC activation in cultured cells. (**a**) Representative *α*-SMA western blots on HSC-T6, LX-2, or primary rat HSCs treated with exogenous t-PA for 24 h, using pooled whole-cell lysates (*n*=2–3/well); quantification on right (*n*=5 independent experiments per cell source). (**b**) Quantifications of western blots for *α*-SMA on lysates from HSC-T6 cells treated for 24 h with protease-inactivated t-PA (*n*=4). (**c**) RT-PCR for *Col1a1* mRNA expression in primary HSCs or HSC-T6 cells after 24 h t-PA treatment; quantification on right (*n*=3 and 4, respectively). (**d** and **e**) HSC-T6 cells stimulated with protease-inactivated t-PA (**d**) or t-PA (**e**) for 48 h before measuring mitochondrial function (MTT reduction assay; *n*=3) or apoptosis (TUNEL labeling). Error bars±s.e.m. *P<0.05, **P<0.01, ***P<0.001, Student's paired (**a** and **b**) or unpaired (**c**–**e**) *t*-test.

To examine the effects of t-PA on HSC phenotype, immortalized HSC cell lines (rat HSC-T6, human LX-2) or primary rat HSCs were treated for 24 h in serum-free conditions with either t-PA (Figure 1a) or non-proteolytically active t-PA (Figure 1b). All t-PAs tested led to a reproducible decrease in *α*-SMA protein expression, a marker of HSC activation. These data were surprising as previous work in collaboration with our laboratory found that t-PA increased *α*-SMA in the kidney fibroblast cell line NRK-49F.^9^ To rule out possible experimental differences, NRK-49F cells were obtained and treated with t-PA in the same conditions as the HSCs. Supplementary Figure S2 shows that t-PA does indeed induce *α*-SMA in NRK cells, indicating the effects of t-PA are cell type-specific. Next, using HSC-T6 cells or primary cells, we verified that t-PA alters other markers of HSC activation, ie, extracellular matrix production. Figure 1c shows that Collagen I mRNA expression in primary HSCs decreases after t-PA treatment. Lastly, we looked for changes in cell metabolism and viability in response to t-PA. No changes in MTT and TUNEL were observed with non-proteolytically active t-PA treatment at 48 h (Figure 1d), although active t-PA did decrease cell viability and increase cell death (Figure 1e). Collectively, these data demonstrate that *in vitro*, t-PA downregulates markers of HSC activation, and active t-PA can further induce moderate cell death.

### The Effects of t-PA on HSC Activation Are Dependent on LRP1-Mediated Signaling

**Figure 2:**
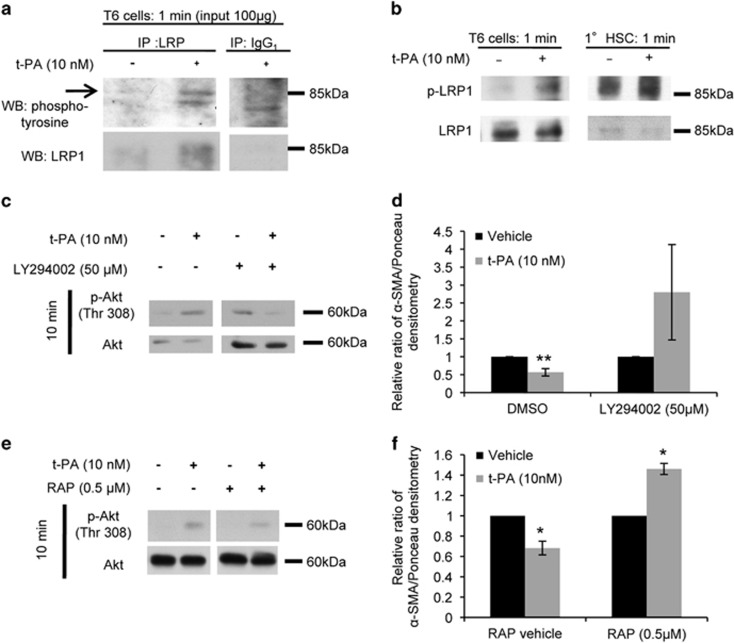
The effects of t-PA on HSC activation are mediated by LRP1-dependent signaling. Whole-cell lysates either immunoprecipitated with anti-LRP1 followed by western blot for phospho-tyrosine and LRP1 in parallel (**a**), or directly analyzed by western blot with antibodies against p-LRP1 and total LRP1 (**b**). (**c** and **d**) HSC-T6 cells pretreated with PI3K pathway inhibitor LY294002 or DMSO for 60 min before co-treatment with t-PA or vehicle for 10 min (**c**) or 24 h (**d**). (**e** and **f**) HSC-T6 cells acid-washed (pH 5) at 4 °C to dissociate endogenous ligands, pretreated with the LRP1 inhibitor RAP or vehicle for 30 min, then co-treated with t-PA or vehicle for 10 min (**e**) or 24 h (**f**). (*n*=6 and 3 for **d** and **f**, respectively.) Panels compared in **a**, **c**, and **e** are noncontiguous lanes from single blots. Error bars±s.e.m. *P<0.05 and **P<0.01, Student's paired *t*-test.

Although plasmin, only generated by active t-PA, is reportedly anti-fibrotic,^21^ both the proteolytically active and inactive forms of t-PA can signal through the LRP1 receptor.^8^ To determine if the observed decrease in HSC activation is mediated through LRP1, HSC-T6 cells were treated with t-PA and protein isolates were analyzed for phosphorylation of LRP1. Following immunoprecipitation with anti-LRP1, an 85-kDa phosphorylated band that migrates at the same molecular weight as the *β*-chain of LRP1 was selectively increased after treatment (Figure 2a). As a second, non-specific phosphorylated band within the same size range was also detected by the immune control in the t-PA-treated samples, to verify the individual band was truly phospho-LRP1, we further probed whole-cell lysates with a phospho-specific antibody against the Tyr4507 residue of LRP1. These results confirm that increased LRP1 phosphorylation was detected within 1 min of t-PA addition in HSC-T6 and primary rat HSCs (Figure 2b).

Phosphorylation of tyrosine 4507 within the LRP1 cytoplasmic domain is essential for LRP1-dependent downstream signaling.^22, 23^ To test whether t-PA acts through known signaling targets of LRP1, the Akt and ERK pathways were examined. Both Akt and ERK were activated in t-PA treated HSC-T6 cells within 10 min post-treatment (Supplementary Figures S3A and B). Inhibition of the ERK pathway was ineffective in changing outcome (data not shown); however, the effects of t-PA on HSC activation were abolished in the presence of the PI3K/Akt pathway inhibitor LY294002 (Figures 2c and d). Interestingly, an increase in total Akt was consistently observed when only LY294002 was present but notably, the two t-PA minus controls displayed correlative values for their p-Akt/Akt ratios.

To further confirm the effects we observed were dependent upon signaling through LRP1, cells were treated with the receptor-associated protein (RAP), a specific inhibitor of ligand binding to LRP1.^24^ RAP pretreatment abrogated both Akt phosphorylation (Figure 2e) and the subsequent suppression of *α*-SMA (Figure 2f) although unexpectedly, t-PA significantly increased *α*-SMA expression when RAP was present. We conclude LRP1 signaling through Akt is necessary for the t-PA-mediated suppression of *α*-SMA expression.

### p-LRP1 and t-PA Co-Localization with *α*-SMA Precedes Resolution of Injury in WT Mice

**Figure 3:**
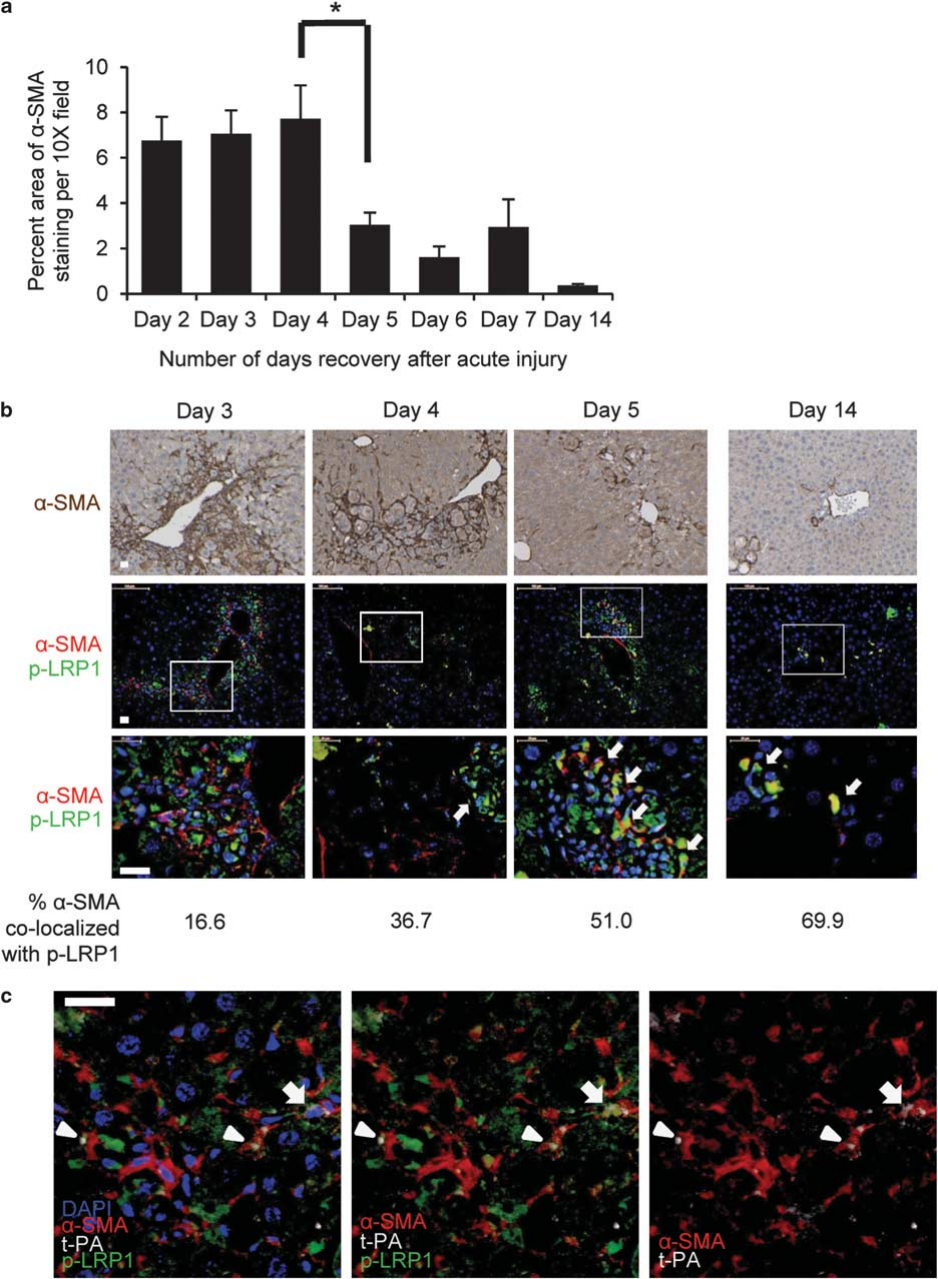
LRP1 is tyrosine phosphorylated in HSCs during resolution of acute hepatic injury *in vivo*. (**a**) Quantification of *α*-SMA immunohistochemistry on tissue sections from WT mice after acute CCl_4_ injury as described in MATERIALS AND METHODS (× 10 images, minimum 10). *P<0.05, one-way ANOVA with post-test comparisons; only sequential differences denoted. Error bars±s.e.m. (**b**) Standard immunohistochemistry (upper panels, *α*-SMA) or double Qdot-labeled immunohistochemistry for p-LRP1 and *α*-SMA (middle and lower panels), with percent *α*-SMA/p-LRP co-localization area of total *α*-SMA staining indicated below each time point. Arrows indicate co-localization. Bottom panels are higher magnifications of boxed regions in the middle panels. Days indicated are post-CCL_4_. (**c**) Qdot-labeled immunohistochemistry for *α*-SMA, t-PA, and p-LRP1 on day 4 post-CCL_4_. Selected channels separated from the same image shown for comparison. Arrow indicates triple co-location, arrowhead indicates *α*-SMA/t-PA co-localization. Scale bars, 20 *μ*m.

As the *in vitro* data indicated that LRP1-mediated signaling decreases HSC activation markers, we hypothesized that t-PA would regulate HSC activation *in vivo* as well. To begin to test this hypothesis, we first determined the timing of HSC regressions during resolution of an acute, *in vivo* injury. WT mice were subject to acute liver injury by CCl_4_ and allowed to recover for up to 14 days. Tissue sections were stained for *α*-SMA which is absent in quiescent mouse HSCs.^1^ As shown in Figure 3a, the *α*-SMA^+^ population drops off significantly between days 4 and 5 after acute liver injury, and if our hypothesis is correct, we anticipate that p-LRP1, primarily absent in resting liver, should be detected on activated stellate cells at this time. Next, co-immunolocalization of p-LRP1 and *α*-SMA was performed, spanning the same time points (Figure 3b). As phosphorylation of LRP is transient and the half-life of *α*-SMA is 72 h,^25^ we expected to only identify a fraction of *α*-SMA-positive cells that were also p-LRP1 positive. Strikingly, at day 3, very little co-localization was observed (16.6%), suggesting the presence of p-LRP only in other hepatic cell types (Figure 3b). At day 4, increased co-localization was apparent (36.7%); however, from day 5 onward, co-localization was readily observed visually and by software analysis, correlating with the time frame of loss of *α*-SMA-positive cells as observed in Figure 3a. The magnified inserts shown in Figure 3b highlight non-vascular *α*-SMA+ regions in order to focus on *de novo* activated HSCs, rather than *α*-SMA+ vascular smooth muscle cells.

As LRP1 is present on several cell types and can be activated by a variety of ligands, sections from WT mice were co-immunolabeled at day 4 of recovery with antibodies against t-PA, p-LRP1, and *α*-SMA. Although sparse, regions were identified where all three proteins were co-localized in addition to several regions with t-PA adjacent to *α*-SMA^+^ cells (arrow and arrowheads, respectively, Figure 3c). This indicates *in situ* proximity of t-PA with activated HSCs just prior to the observed increased in HSC p-LRP1 (day 5; Figure 3b), further implicating t-PA-LRP1 interactions in the resolution of HSC activation during liver repair.

### t-PA- and LRP1-Deficient Mice Retain More *α*-SMA-Positive Cells After Injury

**Figure 4:**
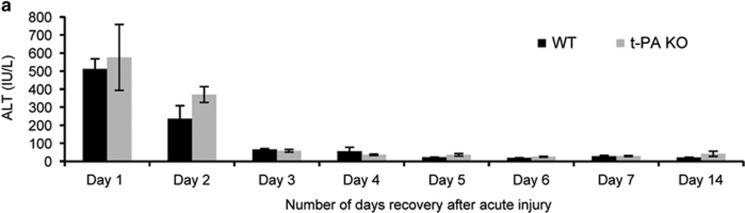

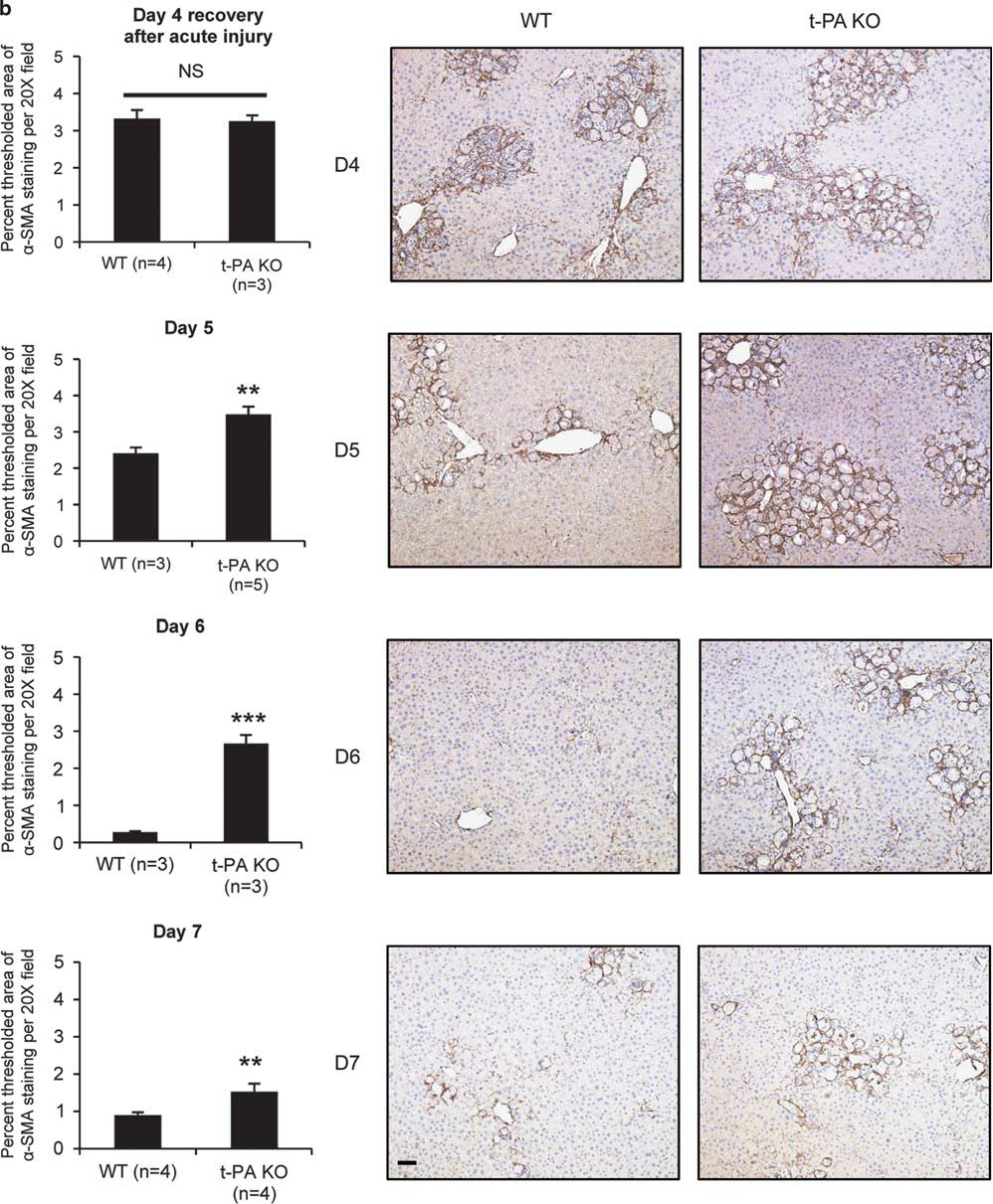
t-PA promotes efficient resolution of acute hepatic injury in mice. C57Bl/6 (WT) and t-PA null mice (t-PA KO) analyzed at days 1–7 and 14 post-CCL_4_ injury as described in MATERIALS AND METHODS. (**a**) Serum ALT values in WT and t-PA KO mice. No values were significantly different at time points indicated. (**b**) Quantification of *α*-SMA staining (× 20 images, minimum 10/animal). Sections from WT and KO mice on matching days were stained, imaged, and analyzed together. Representative images are shown. Error bars±s.e.m. *P<0.05, **P<0.01, ***P<0.001, unpaired Student's *t*-test. Scale bars, 50 *μ*m.

We next examined t-PA null mice^26^ and assessed whether regression of activated HSCs was impaired compared with WT. Although mice lacking t-PA are known to have impaired fibrin clearance and liver repair through day 7 after injury,^4^ HSC activation was previously not examined. Similar to Bezerra *et al*,^4^ no significant differences were detected in the extent of parenchymal injury between WT and t-PA null mice post-injury (Figure 4a); however, t-PA null mice had significantly larger areas of *α*-SMA-positive-staining per visual field, beginning at day 5 when injury starts resolving in WT (Figure 4b). Hence, overall loss of t-PA affects recovery through maintenance of an *α*-SMA-positive cell population.

**Figure 5:**
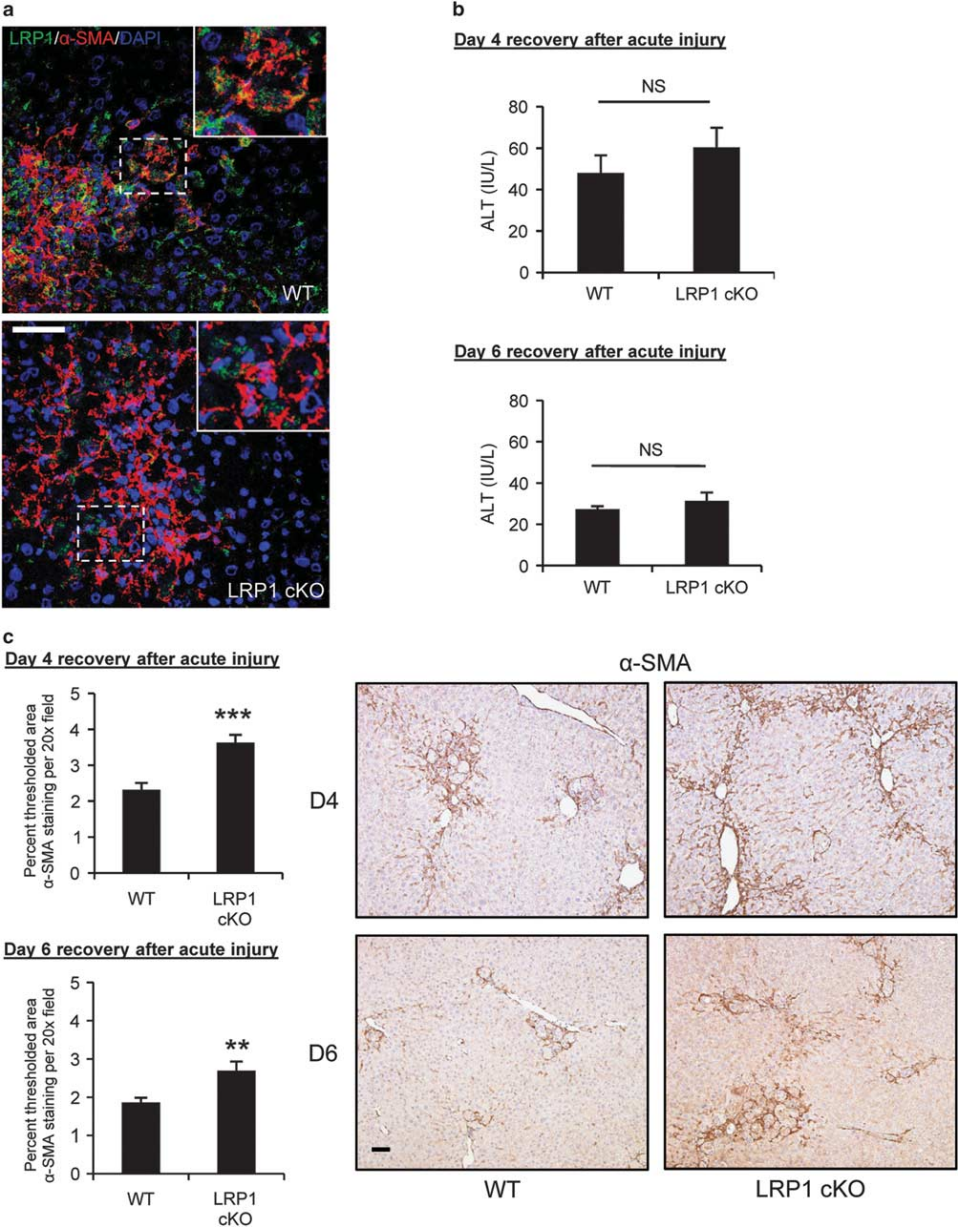

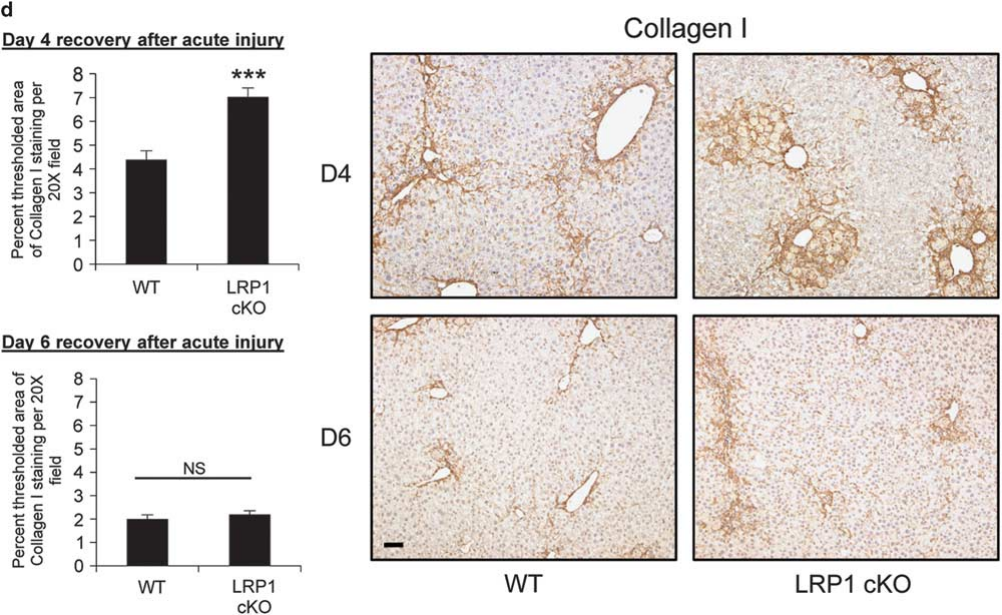
Genetic deletion of LRP1 in HSCs reveals its contribution to resolution of hepatic injury and matrix deposition. *LRP*^*flox/flox*^ (WT) and *LRP*^*flox/flox*^*;SM22-Cre*^*+*^ (LRP1 cKO) mice treated with CCL_4_ as described in MATERIALS AND METHODS. Liver tissue and serum were collected at days 4 and 6 after the last dose. (*n*=3 animals for each time point and genotype). (**a**) Representative confocal images of double immunolabeling with anti-LRP1 and anti-*α*-SMA antibodies of sections from day 4 post-injury. Area inside the dotted squares is shown in the insert. Yellow staining indicates co-localization of LRP1 and *α*-SMA. Scale bars, 50 *μ*m. (**b**) Serum ALT values in WT and LRP1 cKO mice at 4 and 6 days after the last CCL_4_ injection. (**c** and **d**) Representative images (left) and quantification (right) for immunohistochemistry for *α*-SMA (**c**) and Collagen I (**d**) at the indicated time points after injury. Sections from WT and cKO mice on matching days were stained, imaged, and analyzed together. Error bars±s.e.m. *P<0.05, **P<0.01, ***P<0.001, unpaired Student's *t*-test. Scale bars, 50 *μ*m.

As t-PA is globally inactivated in the null mice, we next specifically tested the involvement of LRP1 on activated HSCs by repeating the experiment using conditional LRP1 knockout mice.^27^ SM22, or transgelin, is a smooth muscle cell marker selectively expressed in quiescent and activated HSCs.^28^ Hence, LRP^flox/flox^; SM22-Cre^+^ animals harbor HSCs with a selective deletion for LRP1. Age-matched LRP^flox/flox^; SM22-Cre^+^ mice and genetically matched controls (Cre^−^) were subjected to CCl_4_–treatment and killed at days 4 and 6 post-injury. The loss of HSC LRP1 was confirmed by performing double immunolabeling with LRP1 and *α*-SMA. Although LRP1 remained on other hepatic cell types, a decrease in co-localized signal, relative to total *α*-SMA, was observed at day 4 in the LRP^flox/flox^; SM22-Cre^+^ (LRP1 cKO) mice (Figure 5a). Similar to t-PA null mice, despite no change in parenchymal injury by either day 4 or 6 after injury (Figure 5b), conditional KO mice retained more *α*-SMA^+^ cells than control mice during recovery (Figure 5c). In addition, more Type I collagen was deposited in the tissue of LRP1 conditional KOs at day 4 after injury (Figure 5d). Liver to body weight ratios were not statistically different. Staining for fibrin in the both t-PA and LRP null animals was not different *vs* their wild-type counterparts (see Supplementary Figure S4), similar to the findings of Bezerra *et al.*^4^

## DISCUSSION

HSCs are crucial for development of liver fibrogenesis; their depletion leads to marked amelioration of fibrogenesis and, downregulation of MFB-like cell markers following chronic liver injury.^29^ Hence, developing anti-fibrotic therapies to treat hepatic fibrosis/cirrhosis can potentially decrease morbidity and mortality in patients with chronic liver diseases^30^ who are at risk for hepatocellular carcinoma.^31^ This study identifies the t-PA/LRP1 ligand/receptor pair on activated HSCs as potential new targets for anti-fibrotic therapies.

LRP1 is a multi-functional receptor with a plethora of ligands.^8^ In liver, LRP1 is generally known as a hepatocellular clearance receptor for t-PA.^32^ Although several studies have indicated a role for t-PA during acute injury repair,^4, 5^ little is known with regard to the mechanisms involved, including affected cell types. A recent *in vitro* study demonstrated that LRP1 can mediate anti-proliferative and anti-migratory functions in HSCs.^11^ Our data expands upon that study by (i) identifying t-PA as a signaling ligand for HSC LRP1, (ii) expanding the *in vitro* changes to phenotypic transformation, and (iii) confirming that these effects are relevant *in vivo*.

t-PA is an FDA-approved drug, making it an attractive candidate for anti-fibrotic therapy; however, our findings open up several interesting questions that must first be addressed. First, although proteolytically active and inactive t-PA were both able to signal through LRP1 *in vitro*, only active t-PA was pro-apoptotic (Figures 1d and e). This suggests that *in* vivo, t-PA regulates both populations of activated HSCs described by Kisseleva *et al*,^33^ ie, the cells that die and the cells that revert but remain and are easily reactivated. Targeting cell death with active t-PA may prove challenging, yet inactive t-PA may not prove sufficient. Second, although t-PA-mediated signaling through LRP1 on HSCs decreases their activation, the exact opposite outcome is observed in a model of kidney fibrosis where complex formation between LRP1 and *β*1 integrin^9^ is pro-fibrotic via the ERK pathway.^34^ Notably, we also observed transient ERK activation (Supplementary Figure 3B), although we were unable to block the effects of t-PA through pathway-specific inhibitors (data not shown). Furthermore, t-PA also acted as a pro-fibrotic in our system, but only when binding to LRP1 was prohibited (Figure 2f). Combined, these findings suggest that either there are other LRP1 co-receptors,^9, 35, 36^ other LRP1 ligands, and/or other t-PA receptors^37^ that regulate the outcome. Refined mechanistic studies are still needed to fully parse out the anti- *vs* pro-fibrotic pathways mediated by t-PA and the role of LRP1-mediated signaling in these processes. Finally, the function of t-PA in chronic liver injury remains unclear; two independent studies utilized t-PA null mice in prolonged CCl_4_ fibrosis protocols and reported opposite results.^6, 7^ Interestingly, the longer study (6 *vs* 4 weeks of CCl_4_ treatment) reported less fibrosis in the t-PA null animals as compared with their WT counterparts, despite an increase in liver damage and necrosis. This suggests that extended insults to the liver are invoking important changes in the microenvironment that both alter outcome and are related to t-PA. Importantly, neither chronic study examined the contribution of LRP1 to this process, nor did they examine the ability of t-PA null animals to resolve fibrosis. Hence, a logical next step is to examine the roles of t-PA and LRP1 during resolution of chronic hepatic injury to better determine if t-PA has potential to become a realistic tool for treating patients.
