# Supplementary material for: Tissue-type plasminogen activator suppresses activated stellate cells through low-density lipoprotein receptor-related protein 1
Source: Lab Invest. 2015 Aug 3;95(10):1117–29. doi: 10.1038/labinvest.2015.94 (PMC4586397; doi:10.1038/labinvest.2015.94)
Supplement: Supplementary Figures [file labinvest201594x1.pdf]

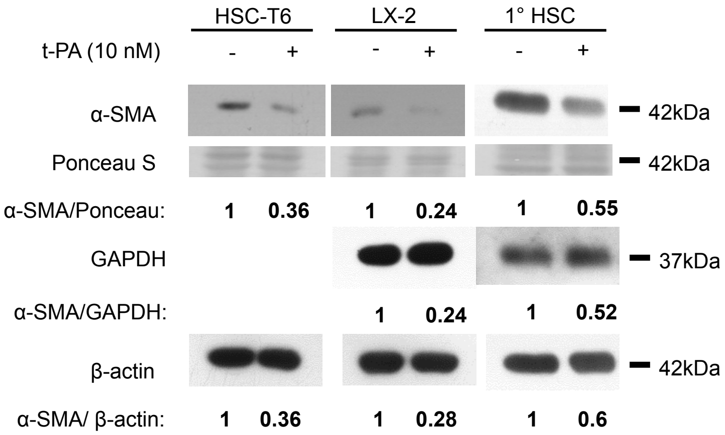

Supplementary Figure S1. Comparison of different loading controls for western blot analyses. The representative western blot analyses of pooled samples, as depicted in Fig. 1A, are shown with their corresponding Ponceau S staining, western blot analyses for GAPDH (when possible), and re-probing for β-actin. After initially probing for α-SMA, blots were stripped and probed for GAPDH and then stripped and assessed for β-actin levels. Densitometry analyses shown below each set of panels represents the α-SMA values normalized for each loading control, relative to treatment with vehicle.

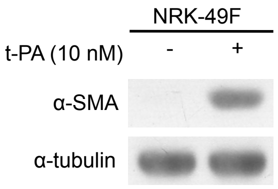

Supplementary Figure S2. Differential effect of exogenous t-PA on kidney fibroblasts. NRK-49F cells were treated with t-PA as described in Materials and Methods. Whole cell lysates were subjected to western blot analysis for α-SMA as a marker of fibroblast activation. The protein loading control α-tubulin is shown.

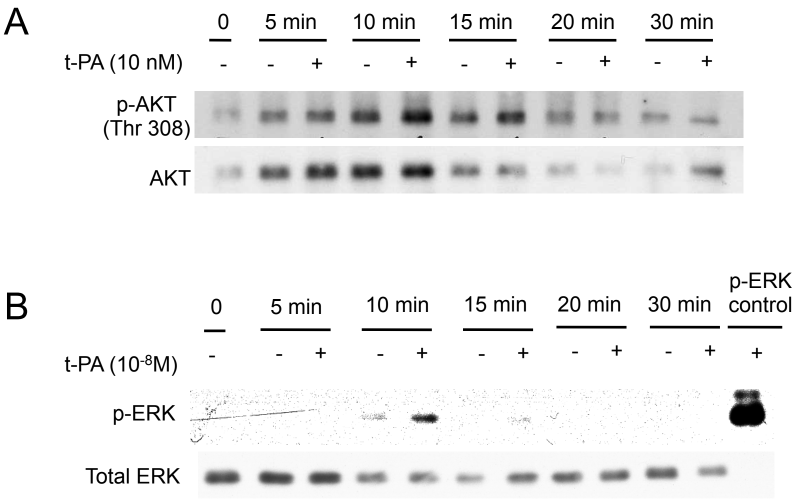

Supplementary Figure S3. Induction of downstream signaling pathways after exogenous t-PA treatment of HSC-T6 cells. HSC-T6 cells were serum starved and treated with t-PA or vehicle control as described in Materials and Methods for varying lengths of time. Whole cell lysates were analyzed by western blot for phospho-AKT and total AKT (A), or phospho-ERK and total ERK (B).

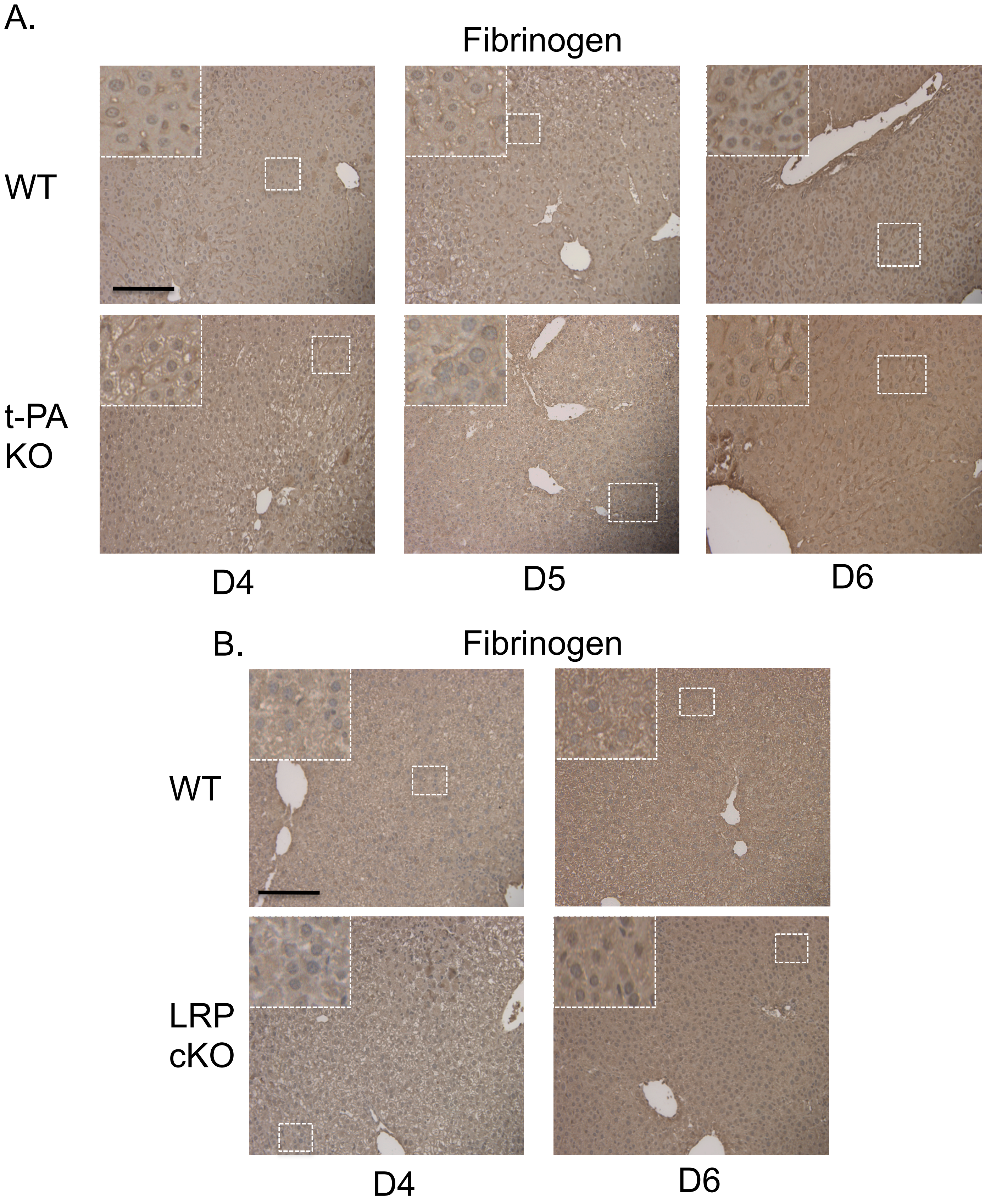

Supplementary Fig. S4. Analysis of fibrin/fibrinogen staining in t-PA and LRP cKOs after acute hepatic injury. **A.** C57Bl/6 (WT) and t-PA null mice (t-PA KO) and **B.** LRPflox/flox (WT) and LRPflox/flox;SM22-Cre+ (LRP1 cKO) were treated with CCL4 as described in Materials and Methods. Representative images of immunohistochemistry with anti-fibrin/fibrinogen antibodies at, A. days 4, 5 and 6 post injury and B. days 4 and 6 post injury. No real differences in fibrinogen staining were detectable in either the t-PA KO or LRP cKO at all time points. Scale bars, 50 μm.
